# Mastocytosis: a rare clinical image

**DOI:** 10.11604/pamj.2023.44.76.36864

**Published:** 2023-02-07

**Authors:** Vaibhavi Chitmulwar, Sourabh Deshmukh

**Affiliations:** 1 Department of Kayachikitsa, Mahatma Gandhi Ayurveda College, Hospital & Research Centre, Salod (H), Datta Meghe Institute of Medical Sciences (DU), Sawangi, Wardha, India

**Keywords:** Mastocytosis, Clonal Bone Marrow Disorder, defective mast cells, dermatographic urticaria

## Image in medicine

Mastocytosis or Clonal Bone Marrow Disorder, is a rare disease characterised by excess of CD34+ mast cell progenitors and functionally defective mast cells. It can affect both children and adults. Hives, itching, and anaphylactic shock are a few of the symptoms that might manifest. Orphan´s Disease is frequently misdiagnosed since it oftenly results from another illness, which means that it might occur more habitually than assumed. This disorder can present in various ways, including cutaneous and systemic mastocytosis. Mast cell´s surfaces display the stemcell factor receptor CD117 (scf). Over 90% of people with systemic mastocytosis have a CD117 gene mutation [KIT (D816V) mutation], resulting in receptor sending out signals continuously. Being a multisystemic condition, it includes a number of symptoms and signs that have an impact on the patient on several levels, including depression, hepatosplenomegaly, dermatographic urticaria, Darier's sign, malabsorption, and stomach discomfort and can be confirmed by biopsy. Antihistamines, cytoreductive treatment (allogeneic stemcell transplantation), and radiation therapy are used to treat the symptoms, which might increase the risk of developing skin cancer. Using this image, one may differently identify carcinoid disease, urticarial vasculitis, autoinflammatory syndrome, mastocytosis, etc. Upon arrival, a 4-year-old girl patient complained of diarrhoea, stomach discomfort, itching and discolouration all over body. Pigmented patches were seen on investigation. The diagnosis of mastocytosis resulted from this, as can be seen in the photo. In Ayurveda, both purifying and comforting treatments were given for three months. After a three-month follow-up, complete remission was seen.

**Figure 1:**
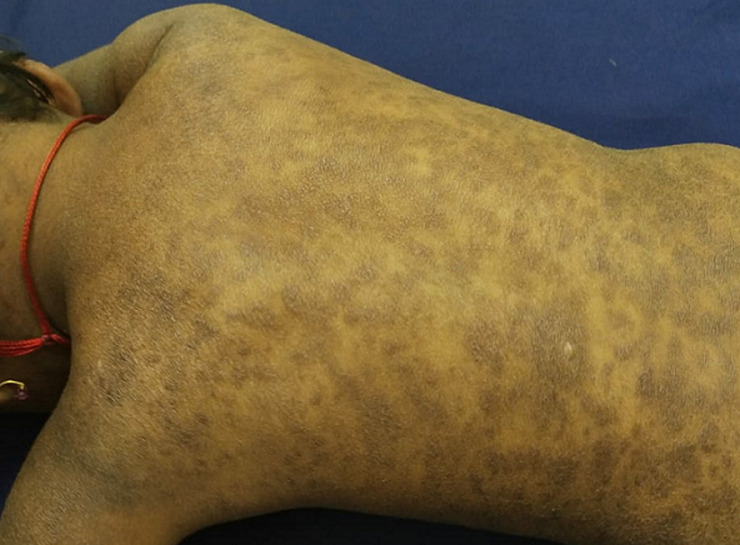
mastocytosis

